# A photoanode with hierarchical nanoforest TiO_2_ structure and silver plasmonic nanoparticles for flexible dye sensitized solar cell

**DOI:** 10.1038/s41598-021-87123-z

**Published:** 2021-04-06

**Authors:** Brishty Deb Choudhury, Chen Lin, Sk Md Ali Zaker Shawon, Javier Soliz-Martinez, Hasina Huq, Mohammed Jasim Uddin

**Affiliations:** 1 Department of Chemistry, PERL-Photonic and Energy Research Laboratory, The University of Texas Rio Grande Valley, 1201 West University Dr, Edinburg, TX 78539 USA; 2 Department of Electrical and Computer Engineering, The University of Texas Rio Grande Valley, 1201 West University Dr, Edinburg, TX 78539 USA

**Keywords:** Electronic devices, Nanophotonics and plasmonics, Photovoltaics, Solar cells

## Abstract

Due to unique photovoltaic properties, the nanostructured morphologies of TiO_2_ on flexible substrate have been studied extensively in the recent years for applications in dye sensitized solar cells (DSSCs). Microstructured electrode materials with high surface area can facilitate rapid charge transport and thus improve the light-to-current conversion efficiency. Herein we present an improved photoanode with forest like photoactive TiO_2_ hierarchical microstructure using a simple and facile hydrothermal route. To utilize the surface plasmon resonance (SPR) and hence increase the photon conversion efficiency, a plasmonic nanoparticle Ag has also been deposited using a very feasible photoreduction method. The branched structure of the photoanode increases the dye loading by filling the space between the nanowires, whereas Ag nanoparticles play the multiple roles of dye absorption and light scattering to increase the light-to-current conversion efficiency of the device. The branched structure provides a suitable matrix for the subsequent Ag deposition. They improve the charge collection efficiency by providing the preferential electron pathways. The high-density Ag nanoparticles deposited on the forest like structure also decrease the charge recombination and therefore improve the photovoltaic efficiency of the cells. As a result, the DSSC based on this novel photoanode shows remarkably higher photon conversion efficiency (η_max_ = 4.0% and η_opt_ = 3.15%) compared to the device based on pristine nanowire or forest-like TiO_2_ structure. The flexibility of the device showed sustainable and efficient performance of the microcells.

## Introduction

Global warming and climate changes are two of the major challenges that the present world is facing currently. Utilization of clean and renewable energy sources (i.e., solar, thermal, wind etc.) has long been an important research area to solve these issues for many decades. Extensive studies and efforts have been applied to the contribution of harvesting clean energy^1^. Solar energy is the most promising one among all the renewable energy sources due to its sustainable, abundant, and readily available nature. Over the past few decades researchers have put significant efforts to improve the performance and stability of the photovoltaic (PV) technologies^2,3^. For example, in the area of inverted metamorphic concentrator solar cells, Geisz et al. proposed that a six-junction (6J) inverted metamorphic multijunction concentrator solar cell design using moderately high junction materials has the potential to exceed 50% efficiency^4^. In PV devices made from silicon, Masuko et al. reported power conversion efficiency (PCE) of 25.6% with crystalline silicon heterojunction solar cell^5^. Currently the market for photovoltaic technology can be broadly divided into two types: (1) large module for terrestrial power supply, and (2) smaller module for powering portable devices. Although PCE is not as high as the above-mentioned works, dye-sensitized solar cells (DSSCs) (PCE usually lower than 10%^2,3^) can play an important contribution to both areas, and have particularly promising potential in the second category^6^. The flexible dye-sensitized solar cells (FDSSC) have emerged as a revolutionary technology in past few years due to their ability to power portable and wearable electronics, especially in applications where flexibility is required^7^. Researchers in this field also strive to maximize PCE for DSSCs. In Zhang et al.’s study, 10.28% PCE of FDSSCs was achieved with a Pt-free counter electrode by sequentially growing polyaniline layers and Co_0.85_Se nanosheets on the surface of carbon fibers^7^. Ardhi et al. implemented a poly(2-ethyl-2-oxazoline) layer at the interface of TiO_2_/N719 dye on the photoanode, with maximum PCE of 11.22%, improved by ~ 32% compared to devices without interlayer^8^. Mathew et al. used molecularly engineered porphyrin dye, coded SM315, to replace N719 dye in regular DSSCs, and achieved maximum PCE value 13.0%^9^.

For a DSSC or FDSSC, the component of critical importance is the dye/metal oxide interfaces. There are various options for the metal oxide film (i.e., TiO_2_, ZnO, SnO_2_, etc.). The major disadvantage to use a nanomaterial with irregular structure as the metal oxide layer is the large number of surface traps, which leads to a series of charge recombination, and slowdown the electron transfer rate^10,11^. Therefore, researchers are particularly interested in adopting well aligned, one dimensional (1D) TiO_2_ nanowire arrays (NWAs) for DSSCs^12–21^. In DSSCs, the vertically grown TiO_2_ NWAs on the surface of the conductive substrate provide straight conducting pathways, resulting in faster charge transport, which is essential for effective charge collection^22–25^. However, one disadvantage of 1D TiO_2_ nanowires is the low surface area because of free space between nanowires. Therefore, development of branched TiO_2_ NWAs has been reported to increase the surface area^26–31^, thus increasing the amount of dye loading and improving light-harvesting property. For example, Shao et al. reported that TiO_2_ nano-tree structure could help to increase the efficiency by ~ 25 to ~ 60%, and the highest PCE (5.45%) was achieved^26^. Sheng et al. also demonstrated that DSSCs based on branched TiO_2_ NWAs showing surface area increasing by 71% and PCE (maximum value 4.61%) increasing by 52%, compared to devices with unbranched NWAs^27^.

Another way to fabricate devices with higher efficiency is to take use of surface plasmon resonance (SPR) from noble metal nanoparticles (NP)^32^. It is well known that when electromagnetic radiation shines on properly fabricated metal nanostructures, the oscillation of confined free electrons in the structure is in resonance with the radiation, resulting in intense, highly localized electromagnetic fields. This phenomenon is called localized surface plasmon resonance (LSPR). Similar to other types of excitation, surface plasmons can decay via a non-radiative pathway via transferring the energy collected from incident radiation to high-energy electrons (hot electrons)^33–36^. Based on different carrier concentrations, sizes and shapes of the nanostructures, hot electrons generated from surface plasmons in Au and Ag nanoparticles can have different energies^37–39^. These hot electrons can be captured when the plasmonic nanostructure is accompanied with an appropriate n-type semiconductor, such as TiO_2_, thus can be widely used in DSSCs. Compared to regular semiconductor-based devices, cells with plasmonic nanostructures are much less affected by the thermodynamic factors. It is comparatively easy to modify the size, shape and composition of the plasmonic nanostructures so that they can absorb wider range of solar spectrum. Besides, plasmonic nanostructures tend to have very strong absorption, which means a high light-trapping efficiency. Recent reports show that incorporating metal NPs, e.g., Au or Ag, on TiO_2_ nanowire surfaces can enhance the light-to-current conversion efficiency^40–45^. For example, Liu et al. incorporated Au nanoparticles in hierarchical TiO_2_ nanorod arrays and the resulted device had PCE value 4.13%^42^. Hu et al. reported Ag NPs synthesized via photoreducing AgNO_3_ on branched TiO_2_ nanorod arrays, and the DSSC with Ag NPs has PCE of 2.83% and exhibits an efficiency improvement by > 50%, compared to devices based on bare branched TiO_2_ nanorods^45^.

**Figure 1:**
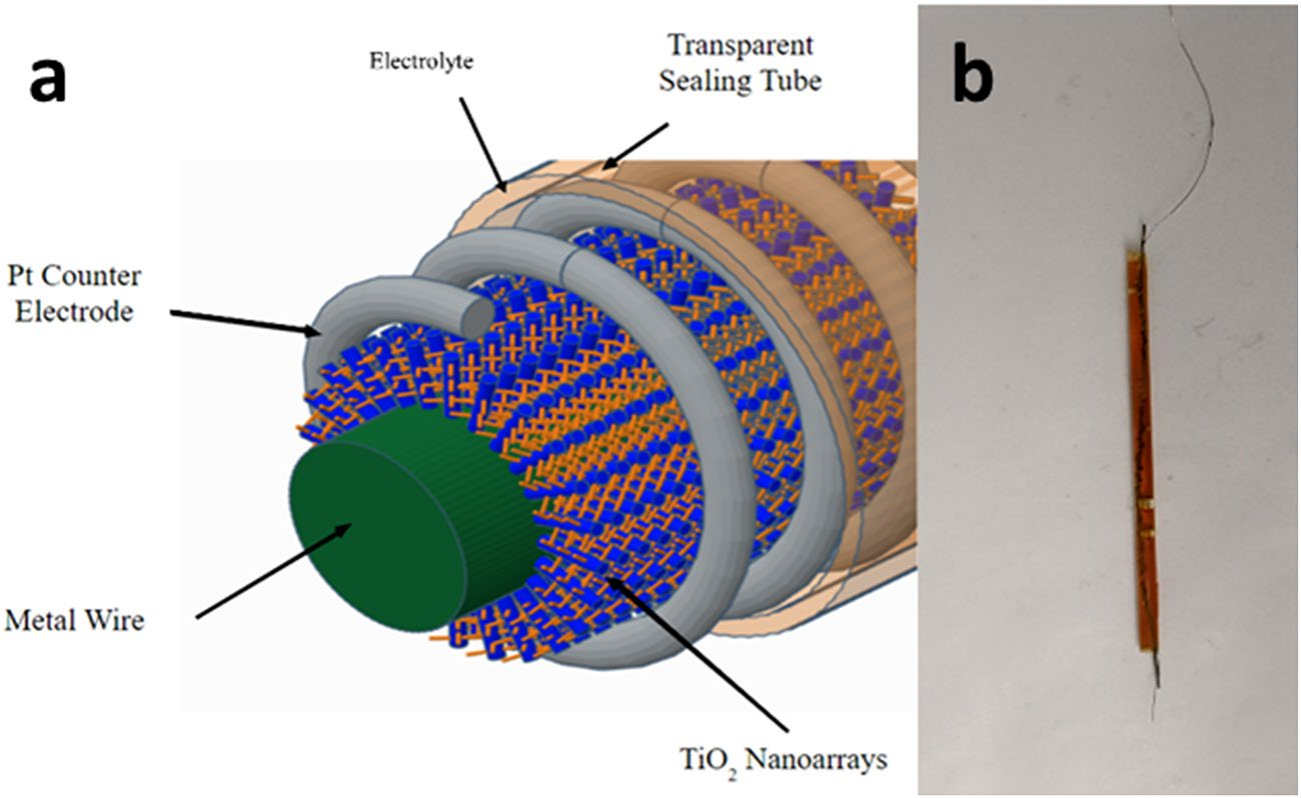
(**a**) Scheme of the device; (**b**) picture of the actual device.

Although most of the above-mentioned works show improved performances of DSSCs, most of them adopt sandwich-shaped flat cell design, i.e., the devices are assembled with the use of fluorine-doped tin oxide (FTO) glass. The rigid substrate forbids the application of DSSCs where flexibility is needed. In order to solve this issue, several groups fabricated FDSSCs with TiO_2_ nanostructures on more flexible substrates such as carbon fibers and Ti wires^20,21,46–48^. However, to the best of our knowledge, there is no report on adopting both directions (synthesis of hierarchical 1D nanostructure and depositing noble metal NPs) at the same time to improve the cell efficiency of FDSSCs based on TiO_2_ nanostructures on Ti wire. In this paper, we fabricated forest-like TiO_2_ branched nanowires on the surface of Ti wire via hydrothermal method. Ag nanoparticles were incorporated in this structure following a very facile photoreduction method^44^. The scheme of the device is shown in Fig. 1. In the SEM images, tree-like nanostructures were observed, and the presence of Ag nanoparticles was confirmed with the EDS data. Through photovoltaic characterization, we showed that the novel FDSSCs fabricated with these photoanodes had better performances than those with bare unbranched 1D TiO_2_ NWAs, possibly because of the improved loading of dye molecules, light scattering, charge transport, and the SPR effect of the metal NPs. Bending test was also conducted to show the flexibility of the device.

## Experimental

### Fabrication of TiO_2_ NWAs

Several Ti wires (ɸ = 250 μm) were cleaned ultrasonically in ethanol and acetone for 15 min, respectively. The treated wires were then put in stainless steel autoclaves half filled with different concentrations (i.e., 0.5 M, 1.5 M, 2.5 M, 5 M, etc.) of NaOH solutions. The sealed autoclaves were put in an electric furnace at 220 °C for a period of 12 h followed by natural cooling to room temperature. The treated nanowires were then covered with Na_2_Ti_2_O_5_∙3H_2_O NWAs. These Ti wires were washed repeatedly with milli-Q water and acetone several times to remove the excess NaOH solution. The wires were then immersed in 1 M to 2 M HCl acid solution for 1 to 2 h for hydrogen ion (H^+^) displacement, and the sample converted to H_2_Ti_2_O_4_(OH)_2_ NWAs.

### Fabrication of nano-tree arrays (NTAs) and decoration with Ag NPs

The as-prepared wires were again transferred to stainless steel autoclaves filled with 25 mL of H_2_SO_4_ solutions (i.e., 0.015 M, 0.02 M, 0.025 M, 0.03 M, etc.). The autoclaves were then put in an electric oven at 100 °C for 4 h. After that, the autoclaves were cooled down to room temperature and the wires were taken out of the autoclaves. The wires were washed repeatedly with milli-Q water and acetone to remove extra acid solution. Then the wires were annealed at 500 °C for 0.5 h to obtain NTAs with improved crystallinity. The as-prepared NTA were then immersed into different concentrations (0.5 mM, 1 mM, 2 mM, etc.) of AgNO_3_ for 5 min and irradiated in UV-crosslinker for 30 min to deposit Ag NPs. The wires were then ready to function as the photoanode.

### Electrolyte preparation

The electrolyte was prepared by dissolving 0.5 M LiI, 0.05 M I_2_, and 0.5 M tert-butyl pyridine and brought up to 10 mL volume in 3-methoxy propionitrile. Poly (vinylidene fluoride-co-hexafluoropropylene) (5 wt%) was added to this solution and the mixture was dissolved overnight with mild heating to make homogeneous.

### Fabrication of fiber-shaped dye sensitized solar cells

The as-prepared photoanodes were immersed into a 0.5 mM N719 dye solution (solvent: mixture of acetonitrile and tert-butyl alcohol in a volume ratio of 1:1) for 24 h. After that, the photoanodes were washed repeatedly with acetonitrile to remove excess dye solution. A platinum wire (ɸ = 125 μm) was twisted around the photoanode and the assembly was put into a transparent capillary tube. The electrolyte was injected into this capillary using a pipette.

### Morphological and photovoltaic characterizations

The morphological characterization was done using scanning electron microscopy (EVO LS10 STEM) equipped with EDS X-ray microanalyzer. The photocurrent–voltage measurements were carried out using a VersaSTAT3 potentiostat (Princeton Applied Research) running cyclic voltammetry with a scan rate of 50 mV∙ s^−1^. A Honle solar simulator 400, with an AM 1.5G spectrum (100 mW/cm^2^) was used to simulate sunlight for irradiating the cells. The PCE (η) was calculated using the equation

$$\eta =\frac{{P}_{\mathrm{m}}}{{P}_{\mathrm{i}}}\times 100\%=\frac{{J}_{\mathrm{m}}\cdot {V}_{\mathrm{m}}}{{P}_{\mathrm{i}}}\times 100\%.$$

In the equation, *P*_m_ is the point on *J*-*V* curve where maximum power density is achieved; *J*_m_ and *V*_m_ are the current density and voltage of the point, respectively; *P*_i_ equals with 100 mW/cm^2^.

## Results and discussion

### Morphology characterization

**Figure 2:**
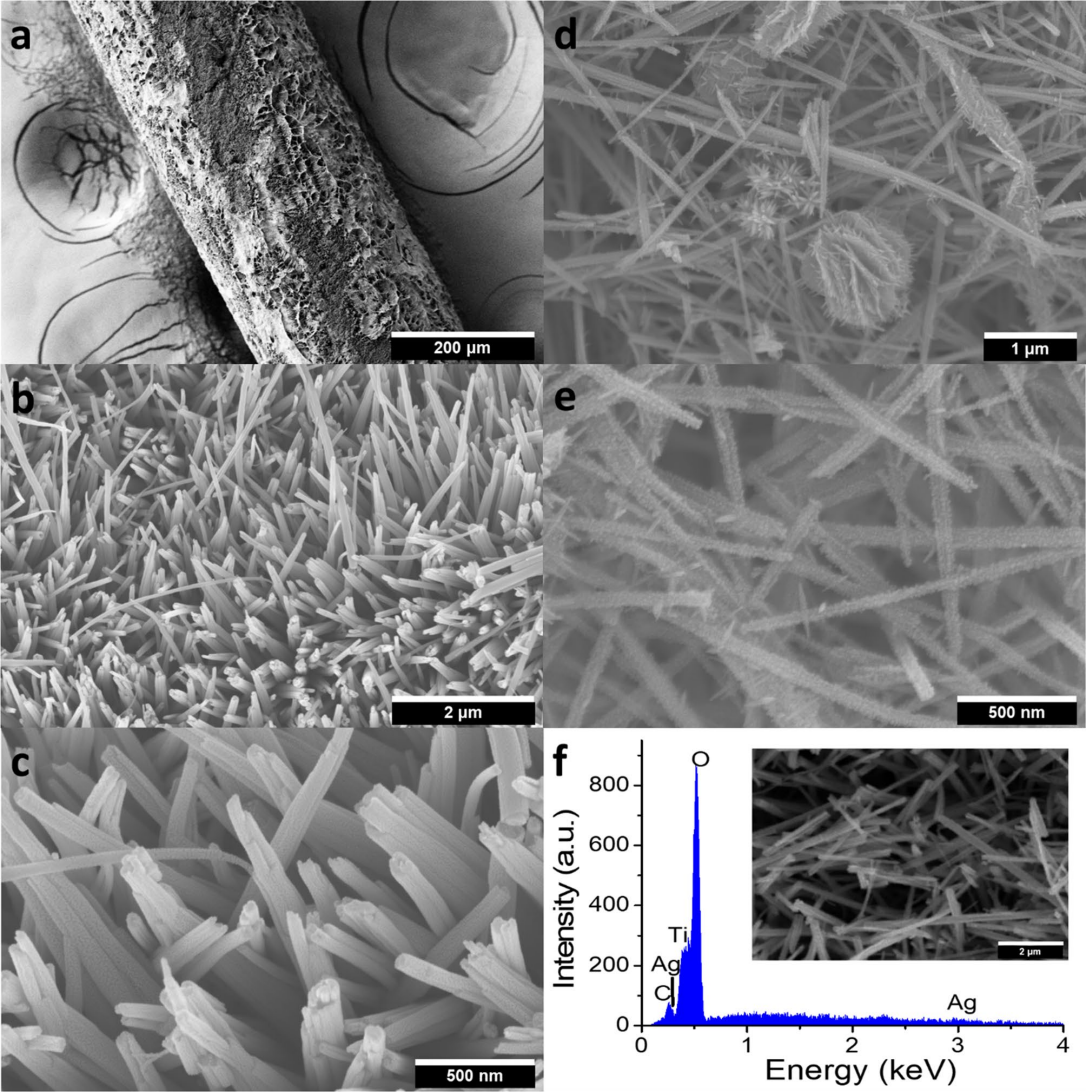
(**a**) SEM image of the Ti thread with TiO_2_ NWAs; (**b,c**) SEM images of TiO_2_ NWAs on the Ti thread; (**d,e**) SEM for TiO_2_ NTAs on the Ti thread at different magnifications; (**f**) EDS analysis of NTA film and the inset is the corresponding SEM image.

The rough surface from Fig. 2a reveals the formation of the NWAs over a large area. The Ti thread becomes uniformly surrounded by the target material. The uniform distribution of the NWAs ensures that no metal surface is exposed and thus inhibits any possibility of short circuiting. Figure 2b,c show the zoomed view of the NWA-covered Ti wire at higher magnifications. It can be seen that the NWs are densely growing on the Ti substrate forming aligned arrays. It can also be seen that the NWs grow vertically and then bend over each other to form a bridged structure. Although we did the photovoltaic performance measurements on the photoanodes prepared with different concentrations of NaOH, a detailed morphological study with varying NaOH concentrations was not carried out in our investigation, as similar works are already available^21,49^. In our investigation, we find a concentration between 2 and 3 M NaOH gives the best morphology. Various parameters play critical roles for the successful deposition of the NWA film (i.e., number of Ti substrates in the hydrothermal reactor, length of the substrates, reaction time, cooling time, etc.). These parameters can significantly change the optimum NaOH concentration for a stable NWA morphology. The reason can be explained by the fact that the number of Ti substrates and their lengths can significantly control the source of Ti wire as well as potential sites for nanowire growth. In our investigation for NTA growth, an optimum concentration of 2.5 M NaOH gives a stable film morphology capable for branched growth. However, a higher NaOH concentration (more than 3 M) always results in cracked films under our experimental conditions, which come out of the substrate, expose bare metals at spots and thus provide potential sites for short circuiting. Figure 2d,e show nano-branches at higher magnifications. Numerous sharp needle-like morphologies with clean and smooth surfaces are clearly visible on individual nanowires. Based on the top view of the SEM images, the needle-like structures have lengths ranging from 19.8 to 47.9 nm. However, it is very interesting that how the differences in H_2_SO_4_ concentrations significantly affect the morphology of the nanostructures. We have treated the nanowires with a range of H_2_SO_4_ concentrations (i.e., 0.015 M, 0.02 M, 0.025 M, 0.03 M, etc.), and have found that an acid concentration of 0.02 M–0.025 M favors formation of needle-like nano-branches, as observed in Fig. 2d,e. After further increasing the acid concentration, e.g., when 0.04 M of sulfuric acid is used, TiO_2_ NTA layers on Ti wires have cracks, and nanowires are found broken into small pieces and aggregates into lumps (Fig. S1). Treating TiO_2_ NWs with sulfuric acid to form branched structures was reported in Shao et al.’s work^26^. Figure 2f shows the EDS spectrum and the corresponding SEM image of the NTA deposited with Ag-NPs. A clear SEM image of Ag NPs on the nano-branches has not been possible to obtain mainly because of the necessity to sputter of gold–palladium (Au–Pd) NPs on them for the measurement. The EDS result confirms the formation of TiO_2_ and deposition of Ag NPs.

### Formation mechanism of NWAs and NTAs

**Figure 3:**
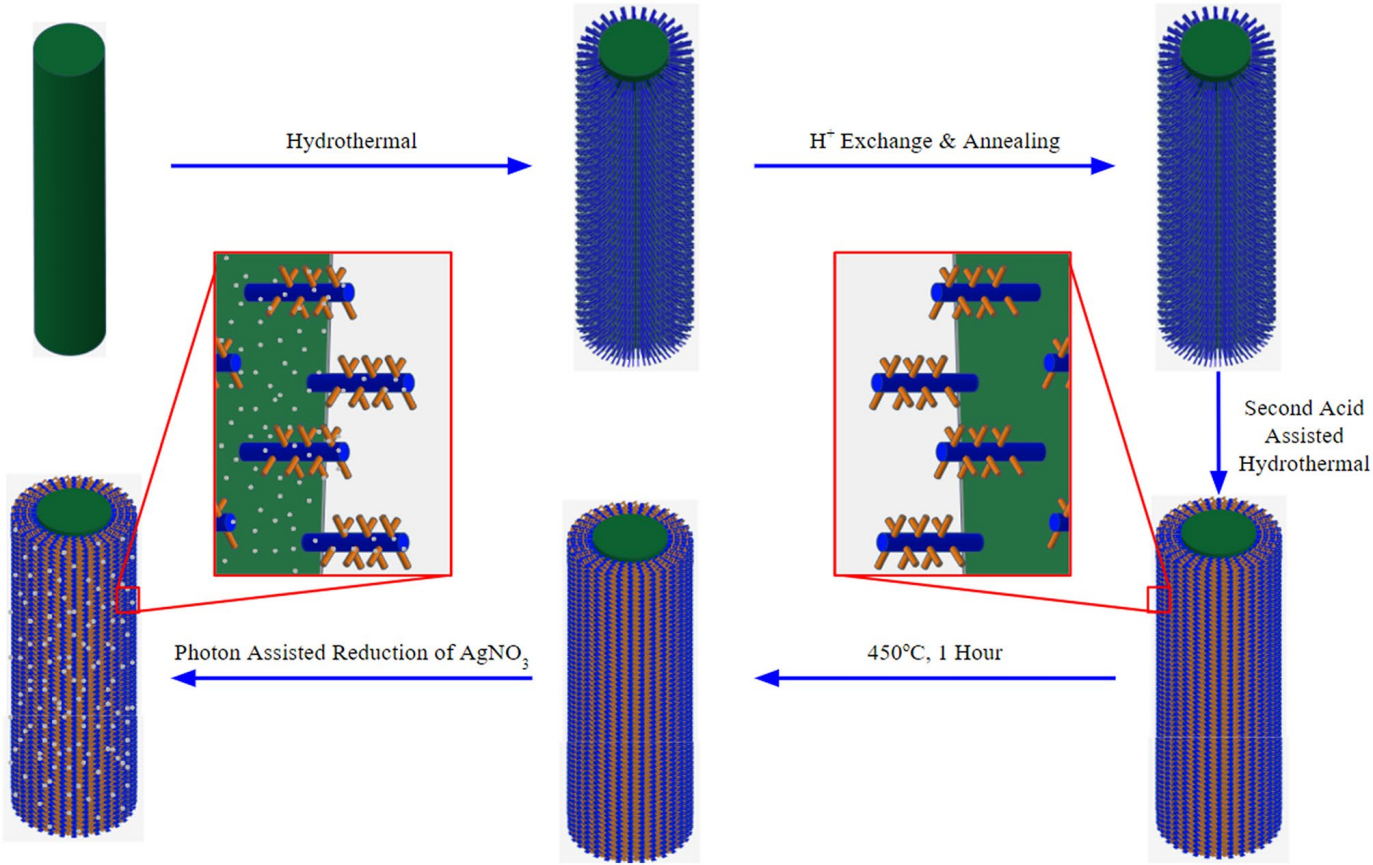
Schematic growth mechanism of the novel hierarchical photoanode.

Scheme of the whole process for Ag-NTA formation is shown in Fig. 3. Shao et al. proposed formation mechanism of NTAs from H_2_SO_4_ hydrothermal reaction to be dissolution–recrystallization process^26^. The Ti wire acts both as the substrate and source of Ti and reacts with NaOH to form Na_2_Ti_2_O_5_·3H_2_O nanocrystals via hydrothermal reaction. The diffusion of Ti atoms from the inside of the wire to the surface initiates the reaction and proceeds with time. The resulted Na_2_Ti_2_O_5_·3H_2_O nanocrystals act as the nuclei for the subsequent growth of the Na_2_Ti_2_O_5_·3H_2_O NWA. Treating NWA with HCl results in the exchange of Na^+^ with H^+^ and forms H_2_Ti_2_O_4_(OH)_2_ NWA. The H_2_Ti_2_O_4_(OH)_2_ NWA reacts with H_2_SO_4_ according to the following reaction:

$$4{{\rm H}_2}{\rm S}{{\rm O}_4} + \, {{\rm H}_2}{\rm T}{{\rm i}_2}{{\rm O}_4}{\left( {\rm OH} \right)_2} \to 2{\rm Ti}{\left( {{\rm S}{{\rm O}_4}} \right)_2} + 6{{\rm H}_2}{\rm O}$$

Ti(SO_4_)_2_ then undergoes hydrolysis to produce TiO_2_ nanoparticles which eventually get deposited onto the H_2_Ti_2_O_4_(OH)_2_ NWA.

$${\rm Ti}{\left( {{\rm S}{{\rm O}_4}} \right)_2} + 2{{\rm H}_2}{\rm O} \, \to {\rm Ti}{{\rm O}_2} + 2{{\rm H}_2}{\rm S}{{\rm O}_4}$$

The success of the process depends on the surface of the NWA, which plays a dual role by providing both the Ti source and locations for crystal growth. Another important parameter is the reaction time. With increasing reaction time, the TiO_2_ nanocrystals gradually forms dendritic structures which eventually transforms into monocrystals^26^. After that, Ag NPs are deposited via AgNO_3_ reduction under UV light.

### Photovoltaic characterizations

**Figure 4:**
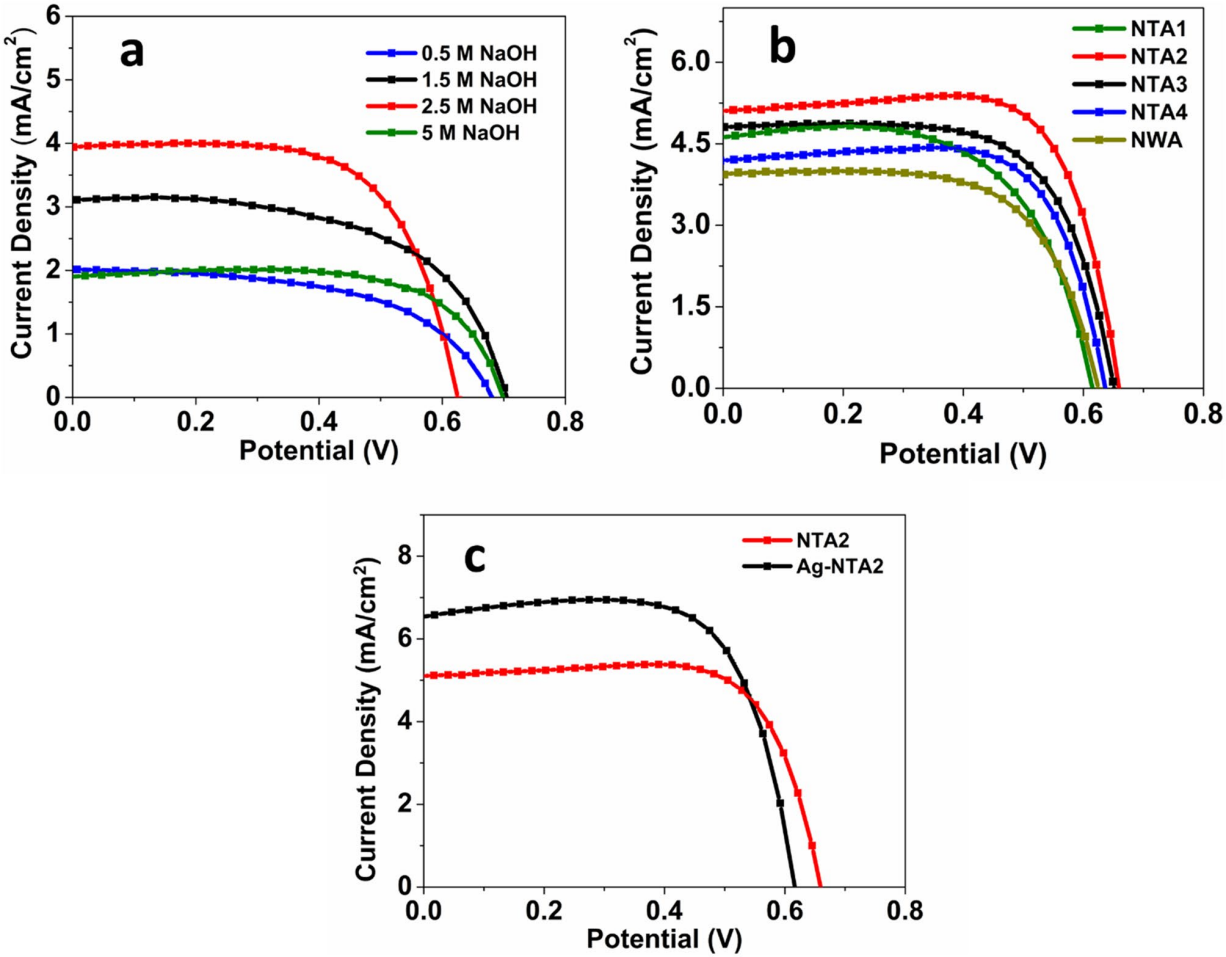
(**a**) Optimization of base concentrations; (**b**) Optimization of H_2_SO_4_ for the NTA; (**c**) Comparison of nano-tree TiO_2_ and Ag deposited nano-tree TiO_2_.

Figure 4a shows the optimization of NaOH concentration to obtain the best performance device under a specific set of experimental conditions. The concentration of NaOH solution has a significant contribution on the length of the NWs, which subsequently affects the light-to-current conversion efficiency. In the past studies, it has been found that the electron diffusion length in the one-dimensional (1D) nanostructures is approximately 100 μm^50,51^. However, in the case of TiO_2_ nanowire, the diffusion length is 10.4 μm^52^. The internal surface area and the amount of dye absorption increase as larger nanowire lengths. As a result, the performance of the solar cell also increases with increasing concentration, as can be seen from Fig. 4a. When the concentration increases from 0.5 to 2.5 M, the efficiency of the cell increases from 0.762 to 1.64%, corresponding to an increase by 115.22%. Higher NaOH concentration not only increases the length of the nanowire but also makes a thicker film. As a result, the amount of dye absorbed also increases^49^. However, an excessively high NaOH concentration, on the contrary, decreases the efficiency significantly, as can be seen in the line for 5 M NaOH solution. The efficiency drops abruptly to 0.974%. With increasing NaOH concentration and hydrothermal reaction time, a continuous film is created at the surface of the Ti substrate by the fusion of the nanowires at their roots due to increased lateral dimension^12,53,54^. As a result of the increased diameter and fusion of the nanowires, the effective internal surface area available for sensitizer trapping is lost, which results in a decreased light-to-current efficiency^55^. The increased diameter and the resulting film reduce the distance that an electron can travel after being injected to the conduction band (CB). When this distance is less than the diffusion length of the nanowire, recombination of the photogenerated electron and hole happens, resulting in the poor performance of the solar cell. At the same time, with a too high NaOH concentration, the NWA film cracks due to the weak contact with the Ti substrate and becomes dispersed powder and thus again reduces the effective internal surface area available. Another important limitation that arises due to the increased NaOH concentration and subsequent increase in nanowire length is the reduced transmittance of the incident light^56,57^. As the nanowire length increases, it blocks the incident light to pass through the inner length and excite the adsorbed sensitizer. As a result, the efficiency decreases. However, surface area of TiO_2_ nanowires is still insufficient compared to other morphologies (i.e., nanoparticles, nanotubes, etc.). To increase the surface area and subsequent dye loading, Fig. 4b shows J–V curves for devices having photoanodes with H_2_SO_4_ corrosion treatment of the nanowires to obtain nano-tree morphology. From Fig. 4a, the optimum concentration of 2.5 M NaOH has been found to be the most efficient under the given set of experimental conditions. A set of acid concentrations (i.e., 0.015 M, 0.02 M, 0.025 M, and 0.03 M) has been used to treat the surface of the nanowires to form branches. Names of corresponding photoanodes have been assigned NTA1, NTA2, NTA3, and NTA4, respectively. Compared with the NWA solar cell, NTA2 gives an increase of 128.08% in the efficiency. This increased efficiency can be attributed to the additional branches that creates more surface area available for the dye absorption. The enhanced light scattering, trapping, fast electron transport offered by the side branches also play significant roles to increase the short-circuit current density (*J*_*sc*_) and PCE in the case of NTA2. The branched structure also allows good penetration of the electrolyte improving the electrode/electrolyte interfacial contact, which in turn improves the fill factor (FF)^58^. The nano-branches also contributes to the improved efficiency by effectively transporting the photoinjected electron from the dye to the electrode. A faster electron transport improves the charge collection efficiency, which in turn improves the short circuit current density from 3.86 to 5.05 mA/cm^2^. The branched structure also provides larger diffusion length and thus inhibits recombination of the photogenerated electrons to holes in the electrolyte. However, as the acid concentration is increased further to 0.025 M, a slight reduction of performance is observed. An increase in acid concentration to 0.03 M significantly decreases the efficiency by 71.0%. The reason can be attributed to the fact that, at higher acid concentration, the nanowires break into individual pieces, probably due to the corrosion of the nanowires. The broken pieces aggregate together to either form lumps or become randomly oriented pieces on the film. The effective surface area reduces significantly, and the number of potential charge trapping sites also increases. This ultimately leads to low charge collection efficiency and hence the poor performance of the device.

To further increase the efficiency, silver nanoparticles were deposited on the NTA to utilize the surface plasmonic resonance (SPR). Figure 4c shows the comparison between bare NTA-based solar cell device and Ag-deposited NTA-based solar cell device. After depositing Ag NPs, the short circuit current density increases significantly from 5.05 to 5.98 mA/cm^2^. As a result, an increase by 18.4% in PCE has been observed. The better performance can be attributed to the reduced recombination of electron–hole pairs due to the Ag-TiO_2_ junction. In the presence of Ag NPs, the absorption range of TiO_2_ NTAs extends in the wavelength region of 400–500 nm due to the SPR. Figure 5 in Hu et al.’s work^45^ showed that absorption in this region is increased after Ag NPs are deposited on branched TiO_2_ nanorod arrays. As a result, the charge carrier concentration increases greatly. When the visible light hits the surface of the Ag NPs, the photogenerated electrons from the Fermi level of Ag jumps to the valance band (VB) of TiO_2_. In the presence of UV irradiation, the photogenerated electrons from the VB of TiO_2_ transfer to the conduction band (CB) of the TiO_2_. The enhanced electromagnetic field that is produced in the presence of Ag NPs improves light absorption of dye molecules and thus excites more electrons than usual^45^. Ag NPs also improve the light scattering due to large plasmonic size^37^. A Schottky barrier is established at the Ag/TiO_2_ junction due to the large work function of Ag. This barrier creates an electric field which promotes the efficient separation of the photogenerated electrons and holes. As a result, the electrons can efficiently travel through the branches and reach the electrode while the hole travels to the electrolyte and get captured by the reduced species^59^. Another way to explain the role of Ag NPs is through the energy levels of the photoanode materials. Under irradiation, the lowest unoccupied molecular orbital (LUMO) of dye molecule contains photogenerated electrons, which can then move to both Ag NPs and the CB of the TiO_2_ simultaneously. Due to the upcoming electrons, the Fermi level of the Ag shifts towards more negative potential until it comes closer to the Fermi level of the TiO_2_. The electrons in Ag NPs then move to the CB of TiO_2_ due to the SPR effect, while the Schottky barrier at the Ag/TiO_2_ junction inhibits the backward diffusion of electrons to the dye or the electrolyte. As a result, the recombination of electrons and holes is significantly reduced, and the efficiency is increased^41^. A comparison of the photovoltaic performance of DSSC based on different photoanodes at standard conditions are shown in Table 1, while Table 2 shows the best photovoltaic performances of DSSCs based on different photoanodes. The corresponding J–V plots for best photovoltaic performance are shown in Fig S2.

**Figure 5:**
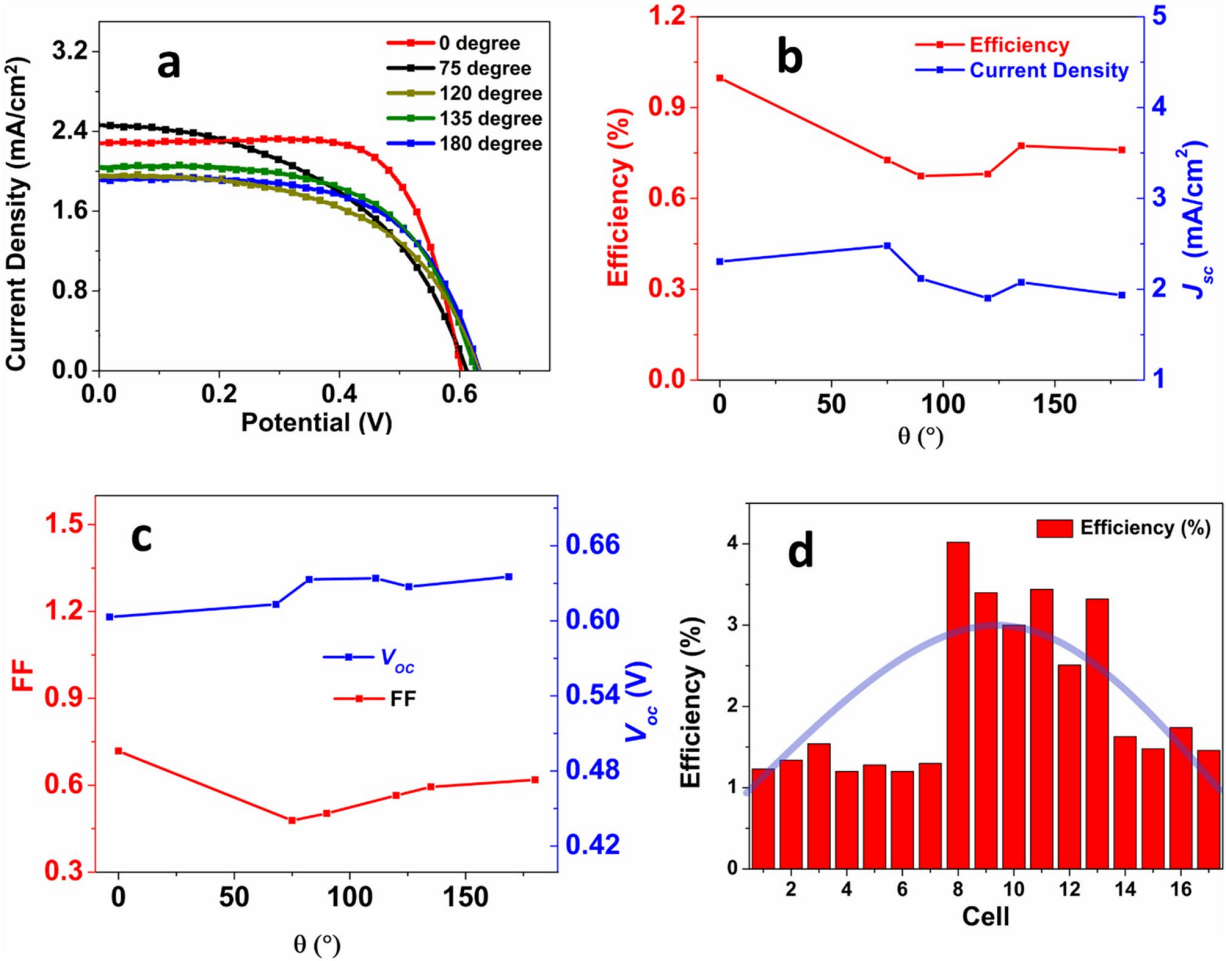
(**a**) The flexibility test of the cells; (**b**) Variation of ƞ and *J*_*sc*_ with bending angle; (**c**) Variation of FF and *V*_*oc*_ with bending angle; (**d**) Efficiency variation of 18 cells.

**Table 1:** Photovoltaic performance of DSSCs based on various photoanodes at standard condition.

Photoanode	*J*_*sc*_*,* mA/cm^2^	*V*_*oc*_*,*Volt	Efficiency, %	FF
NWA	3.86	0.625	1.64	0.68
NTA1	4.63	0.620	1.82	0.64
NTA2	5.05	0.660	2.55	0.77
NTA3	4.75	0.650	2.12	0.69
NTA4	4.18	0.630	1.99	0.75
Ag-NTA4	6.53	0.620	2.96	0.734

**Table 2:** Best photovoltaic performance of DSSCs based on various photoanodes, figure provided in the supporting information (SI).

Photoanode	*J*_*sc*_*,* mA/cm^2^	*V*_*oc*_*,*Volt	Efficiency, %	FF
NWA	2.85	0.713	1.46	0.72
NTA1	4.85	1.03	2.51	0.50
NTA2	5.66	1.11	3.33	0.53
NTA3	5.41	0.890	3.01	0.63
NTA4	2.15	0.730	1.00	0.64
Ag-NTA4	6.41	0.882	4.02	0.71

To test the applicability of the device, various tests have been performed. Figure 5a shows the J–V plot of the cells when they are bent at certain angles. There are a few parameters which can affect the performance of a solar cell during the bending test: (1) electrical property degradation of the Ti wire substrate, (2) electrocatalytic property degradation of the Pt counter electrode, (3) leak of electrolyte, and (4) failure of the TiO_2_ NWAs. It has been found that the efficiency drops only by 28.0% when the bending angle is 75°, making the device suitable for applications that requires flexibility. After that, the efficiency does not vary much even when the bending angle is further increasing. When the bending angle is 180°, the decrease in efficiency is 25%, which is very similar to the initial drop in the performance. The initial drop in efficiency can be explained by above mentioned parameters. However, in actual experiments, no significant leakage of the electrolyte has been found. Past studies have shown that the electrocatalytic performance of platinum counter electrode is not affected by repeated bending^60–62^. As the resistance of Ti substrate does not change significantly upon bending, the reason for decreased performance can be attributed to the failure of the TiO_2_ NTA film. TiO_2_ NTA film cracks and falls off upon repeated bending and thus the efficiency of the device drops initially. Figure 5b shows the relation of efficiency and *J*_*sc*_ with respect to the bending angle. Both *J*_*sc*_ and efficiency show very weak dependence on bending angle. After the cell is bent larger than 75°, there is almost no change observed in *Jsc*. The trend is retained until bending angle reaches 180°. However, efficiency only decreases slightly when the bending angle is 75° and then does not vary much with the bending angle. As already mentioned, failure of the NTA film is the major reason for the decreased performance. Figure 5c shows the behavior of FF and *V*_*oc*_ as functions of bending angle. The FF initially decreases by 33.8% and then keeps increasing slightly with the bending angle. This trend is very understandable from the J–V plot (Fig. 5a), which shows that the maximum power point initially decreases and then increases with bending angle and thus affects the FF. When the cell is bent initially, a disruption is introduced in the nanoscale environment, which probably lowers the maximum power point. However, after initial bending, the disruption stabilizes, and the FF also increased subsequently. The *Voc* stays almost same with the bending angle, which proves that the cell resistance is very stable and does not disrupt charge transport upon bending. This again explains why after initial bending to 75° angle, the *Jsc* value does not change significantly. Figure 5d shows the bar diagram of the efficiency of 18 cells. While most cells have efficiencies close to ~ 1.5%, a significant number of them has efficiency higher than 2.5% with the highest efficiency being 4.02%. A thorough investigation can be done on the individual parameters (length of the substrate, reaction time, connection to the electrode, etc.) to obtain a more consistent trend. With improved connectivity and proper execution of each reaction step, it is very possible to get efficiency significantly higher than 4%.

**Figure 6:**
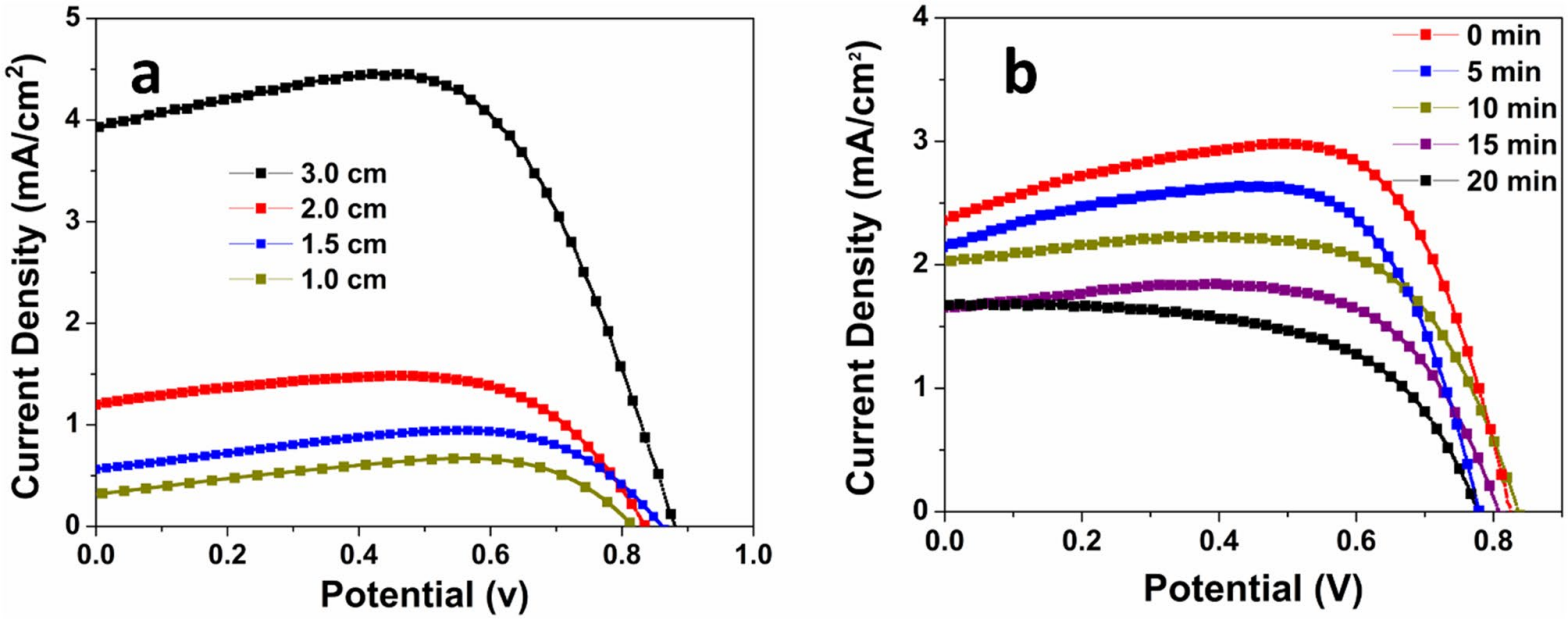
(**a**) Comparison of photovoltaic performances with increasing length; (**b**) Comparison of performances in cold ambient temperature.

Figure 6a shows the effect of cell length on the performance of the device. As expected with increasing length, the performance of the device increases significantly, making it a promising technology for large scale industrial applications, as well as wearable and portable electronics. The efficiency increases from 0.37 to 2.45% when the cell length is increased from 1.0 to 3.0 cm. The increased surface area, higher dye loading, potential site for reduction, and light scattering are the reasons for the higher performance at increasing length. Figure 6b shows the effect of cold temperature in the performance of the device. All three parameters, short circuit current density *J*_*sc*_, open circuit voltage *V*_*oc*_, and PCE decrease with increasing time in cold environment (thus decreasing temperatures), which is very understandable from the fact that, charge transport increases at higher temperature and decreases at lower temperature. However, the cell still works at a reasonable efficiency at lower temperature, making it suitable for applications requiring diverse operational temperatures.

## Conclusion

In summary, fabrication of a hierarchical photoanode with TiO_2_ nano-tree arrays and plasmonic Ag NPs for FDSSCs has been reported. A two-step facile hydrothermal route has been adopted to obtain the desired TiO_2_ morphology and a photon-assisted and solution processed synthesis route has been used to deposit Ag nanoparticles. The device using the resultant photoanode gave 16.1% increase in efficiency compared to bare NTA based device and 81.6% increase in efficiency compared to the corresponding NWA based device. A relative comparison of the performance obtained from devices based on three different types of photoanodes has been performed. Optimization of different parameters has also been done. The higher internal surface area, improved dye loading and light scattering, SPR effect of Ag NPs have been identified as reasons behind the higher performance of the novel photoanode among many other reasons. To test the applicability of the device, bending test, comparison with length, and duration in cold environment have also been performed. Despite a slight drop in the performance, all three tests point out towards a variety of application where the photoanodes can be suitable. Further study in this area can be done to better understand the experimental and operational variables. For example, a study could be conducted to find out the optimum concentration of AgNO_3_ solution for depositing Ag NPs. A thorough investigation on the morphological characterization of the nano-tree arrays and size dependent gradient distribution of the Ag NPs could also be done.

## Supplementary Information

Supplementary information.
