# Supplementary material for: A photoanode with hierarchical nanoforest TiO2 structure and silver plasmonic nanoparticles for flexible dye sensitized solar cell
Source: Sci Rep. 2021 Apr 6;11:7552. doi: 10.1038/s41598-021-87123-z (PMC8024298; doi:10.1038/s41598-021-87123-z)
Supplement: Supplementary file 1 — Supplementary information. [file 41598_2021_87123_MOESM1_ESM.docx]

**A photoanode with hierarchical nanoforest TiO_2_ structure and silver plasmonic nanoparticles for flexible dye sensitized solar cell
Supporting Information**

Brishty Deb Choudhury, Chen Lin, Sk Md Ali Zaker Shawon, Javier Soliz-Martinez, Hasina Huq,^1^ M. Jasim Uddin*

Department of Chemistry, *PERL-Photonic and Energy Research Laboratory*, The University of Texas Rio Grande Valley, 1201 West University Dr, Edinburg, TX 78539, United States

^1^Department of Electrical and Computer Engineering, The University of Texas Rio Grande Valley, 1201 West University Dr, Edinburg, TX 78539, United States

*Corresponding Author Email: mohammed.uddin@utrgv.edu

**
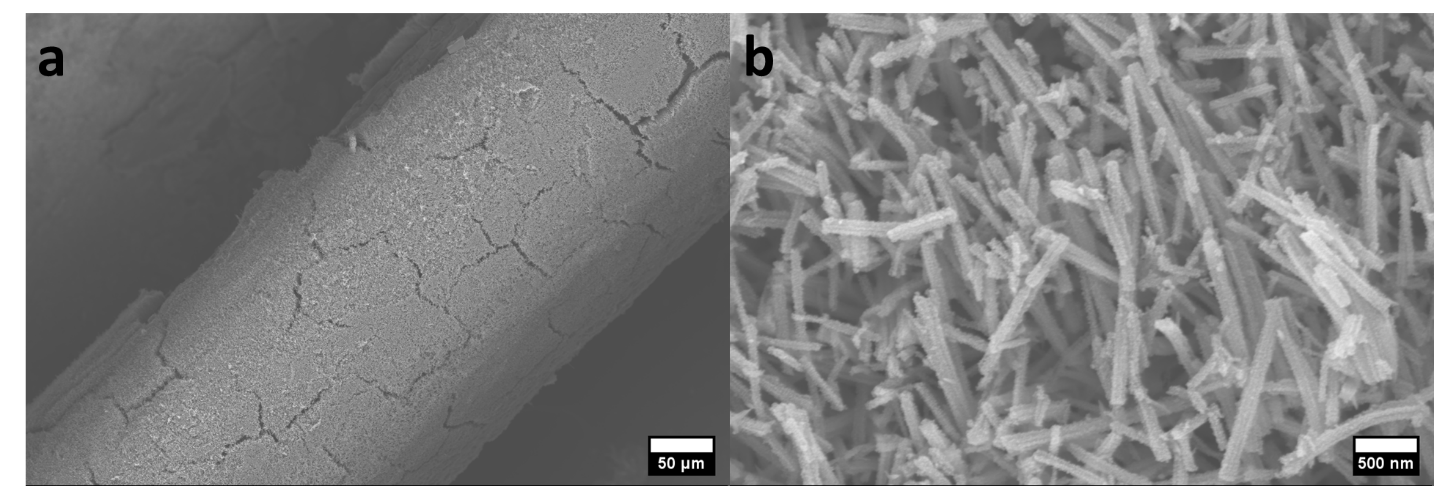
**

**Fig S1. a)** SEM image of the Ti wire with TiO_2_ NTAs after treating with 0.04 M H_2_SO_4_; **b)** SEM image of TiO_2_ NTAs after treating with 0.04 M H_2_SO_4_.


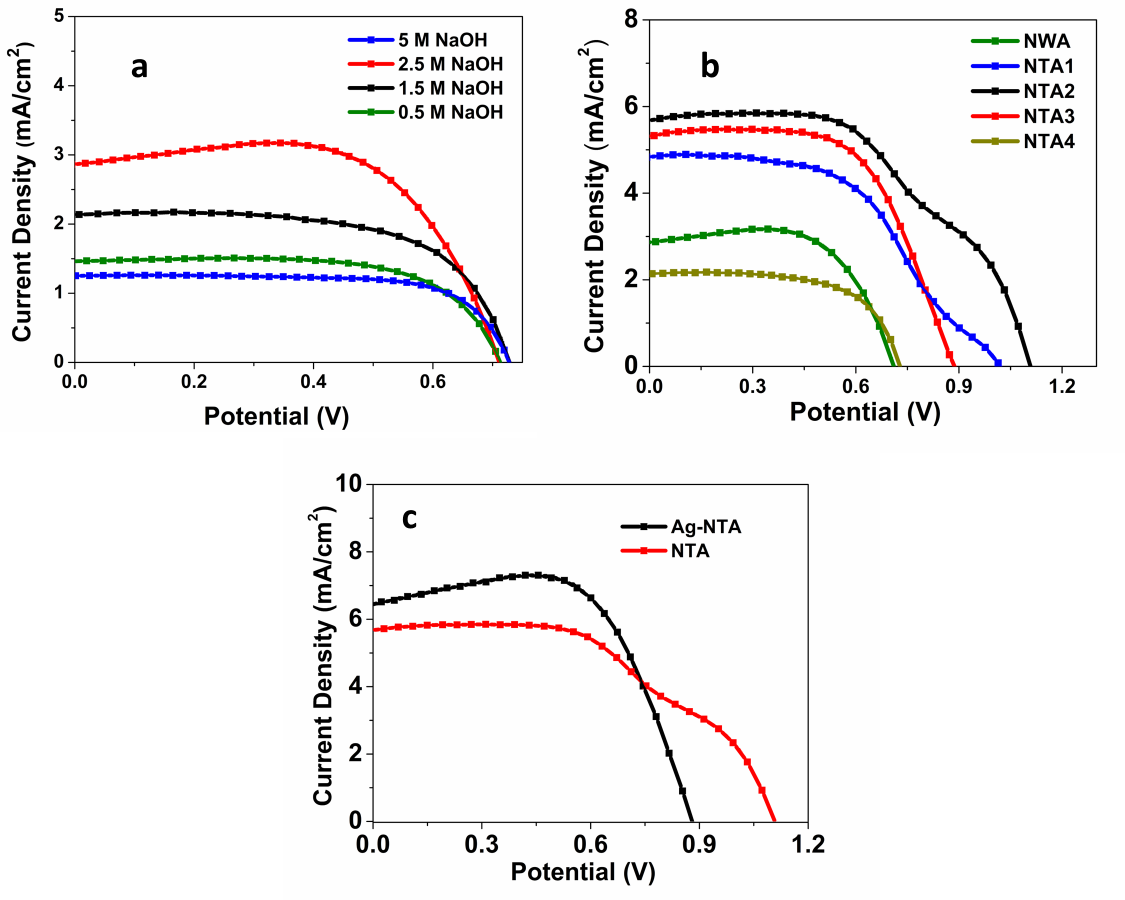


**Fig S2.** I-V curves for best performed photoanodes after optimization in H_2_SO_4_ concentration and Ag deposition (Based on Table:2). **a)** optimization of NaOH concentration for TiO_2_ nanowire growth; **b)** optimization of H_2_SO_4_ concentration for branched TiO_2_ nanowire growth; **c)** comparison of performance between TiO_2_ nano-tree and Ag deposited TiO_2_ nano-tree based devices.
